# A radiogenomics biomarker based on immunological heterogeneity for non-invasive prognosis of renal clear cell carcinoma

**DOI:** 10.3389/fimmu.2022.956679

**Published:** 2022-09-13

**Authors:** Jiahao Gao, Fangdie Ye, Fang Han, Haowen Jiang, Jiawen Zhang

**Affiliations:** ^1^ Department of Radiology, Huashan Hospital, Fudan University, Shanghai, China; ^2^ Department of Urology, Huashan Hospital, Fudan University, Shanghai, China; ^3^ Fudan Institute of Urology, Huashan Hospital, Fudan University, Shanghai, China; ^4^ National Clinical Research Center for Aging and Medicine, Huashan Hospital, Fudan University, Shanghai, China

**Keywords:** clear cell renal cell carcinoma, radiogenomics, tumor heterogeneity, immune microenvironment (IME), contrast-enhanced computed tomography (CECT)

## Abstract

**Background:**

Tumor immunological heterogeneity potentially influences the prognostic disparities among patients with clear cell renal cell carcinoma (ccRCC); however, there is a lack of macroscopic imaging tools that can be used to predict immune-related gene expression in ccRCC.

**Methods:**

A novel non-invasive radiogenomics biomarker was constructed for immune-related gene expression in ccRCC. First, 520 ccRCC transcriptomic datasets from The Cancer Genome Atlas (TCGA) were analyzed using a non-negative matrix decomposition (NMF) clustering to identify immune-related molecular subtypes. Immune-related prognostic genes were analyzed through Cox regression and Gene Set Enrichment Analysis (GSEA). We then built a risk model based on an immune-related gene subset to predict prognosis in patients with ccRCC. CT images corresponding to the ccRCC patients in The Cancer Imaging Archive (TCIA) database were used to extract radiomic features. To stratify immune-related gene expression levels, extracted radiogenomics features were identified according to standard consecutive steps. A nomogram was built to combine radiogenomics and clinicopathological information through multivariate logistic regression to further enhance the radiogenomics model. Mann–Whitney U test and ROC curves were used to assess the effectiveness of the radiogenomics marker.

**Results:**

NMF methods successfully clustered patients into diverse subtypes according to gene expression levels in the tumor microenvironment (TME). The relative abundance of 10 immune cell populations in each tissue was also analyzed. The immune-related genomic signature (consisting of eight genes) of the tumor was shown to be significantly associated with survival in patients with ccRCC in TCGA database. The immune-related genomic signature was delineated by grouping the signature expression as either low- or high-risk. Using TCIA database, we constructed a radiogenomics biomarker consisting of 11 radiomic features that were optimal predictors of immune-related gene signature expression levels, which demonstrated AUC (area under the ROC curve) values of 0.76 and 0.72 in the training and validation groups, respectively. The nomogram built by combining radiomics and clinical pathological information could further improve the predictive efficacy of the radiogenomics model (AUC = 0.81, 074).

**Conclusions:**

The novel prognostic radiogenomics biomarker achieved excellent correlation with the immune-related gene expression status of patients with ccRCC and could successfully stratify the survival status of patients in TCGA database. It is anticipated that this work will assist in selecting precise clinical treatment strategies. This study may also lead to precise theranostics for patients with ccRCC in the future.

## Introduction

Kidney cancer is one of the most common urological tumors, with the number of new patients with renal cancer reaching up to 90,0000 each year (1, 2). Clear cell renal cell carcinoma (ccRCC) is the most common pathological subtype of kidney cancer, accounting for 70%–80% (3, 4) of renal cancers. Early surgical intervention is currently the primary treatment for ccRCC (5, 6). Most patients who undergo early resection have an overall 5-year survival rate of >90%. Some patients with ccRCC have extremely high rates of recurrence and metastasis, which severely affects postoperative survival (7, 8). Some targeted therapies have shown decent treatment effects in ccRCC patients, including sorafenib and axitinib (9, 10). However, the indications for the application of targeted drugs remain highly controversial (11, 12). Therefore, there is an urgent need for non-invasive indicators to effectively diagnose ccRCC patients with different therapeutic reactions, thus enabling rational selection of clinical ccRCC treatment strategies.

Tumor heterogeneity is closely associated with the significant prognostic variability of current tumor therapies in ccRCC patients (13, 14). The synergy between tumor cells and the microenvironment is an important factor in tumor heterogeneity (15, 16). Immune and stromal cells, which represent important components of the TME, are considered to be closely related to the aggressiveness and the developmental potential of ccRCC. The heterogeneous expression of immune-related genes is thought to correlate with ccRCC prognosis (17, 18). Therefore, the exploitation of immune-related prognostic markers is considered an important tool to improve the diagnosis and treatment of ccRCC. Radiogenomics combines gene expression information with medical imaging features, thus enabling an in-depth understanding of tumor biology and capture of internal tumor heterogeneity information (19, 20). Traditional genetic analysis of the tumor based on invasive biopsy is costly and cannot fully reflect the heterogeneity of tumor microenvironment (TME) (21). Radiogenomics has the potential to become a promising non-invasive diagnostic method that can reflect gene expression information (22).

In this study, we constructed a novel radiogenomics method based on TME-related gene profiles. The immune-related gene expression risk score was calculated and predicted using the radiogenomics approach to build molecular markers for the non-invasive prognosis evaluation of ccRCC. Such a strategy may assist in making precise clinical treatment decisions and achieving precise theranostics for ccRCC.

## Materials and methods

### Data processing

Transcriptomic data and relevant clinical and pathological information were extracted from The Cancer Genomics Atlas (TCGA) for ccRCC patients, with a total of 539 samples. To obtain reliable conclusions, samples with a <30-day survival rate were excluded, leaving 520 ccRCC samples enrolled in the downstream analysis. In addition, immune-related (IR) gene symbol names were obtained from the Immunology Database and Analysis Portal (ImmPort). The corresponding enhanced CT digital images were acquired from The Cancer Imaging Archive (TCIA) database. Initially, we collected 267 digital images, and some CT images were excluded based on set criteria for image collection (poor image quality or failure to identify the area of the lesion by the imaging physicians).

### Identification of ccRCC subtypes

The obtained TME-related genes were used for non-negative matrix decomposition (NMF) clustering to identify ccRCC molecular subtypes, and the optimal cluster number K value was determined to be 2. Non-negative matrix factorization (NMF) is an unsupervised learning algorithm that extracts available features. NMF works similar to the principal component analysis and can be employed for dimensionality reduction. Principal component analysis (PCA) was performed to determine the robustness and reliability of ccRCC molecular subtypes.

### Investigation of immune cell nfiltration status

To evaluate the status of immune cell infiltration in the TME, the R package “MCPcounter” was used to manage the relative abundance of 10 immune cell populations in each tissue according to the transcriptome data. The cell types included T cells, CD8+ T cells, cytotoxic lymphocytes, B lineage cells, NK cells, monocytic lineage cells, myeloid dendritic cells, neutrophils, endothelial cells, and fibroblasts. Wilcoxon rank-sum test analysis was performed to assess the differences in immune cell infiltration among the distinct molecular subtypes.

### Construction and validation of the risk model

To quantify immune-related correlation patterns for individual tumors, we divided the patients into training and validation groups in a ratio of 7:3, and univariate Cox proportional hazard regression was conducted to identify immune-related prognostic markers. We further applied significant factors to Least Absolute Shrinkage and Selective Operator (LASSO) and univariate Cox proportional hazard regression analyses to construct the risk model. The risk score was calculated based on the coefficients of the candidate genes. According to the median risk score, patients were divided into low- and high-risk groups. To improve the accuracy and practicability of the clinical predictive model, we constructed a nomogram model that included the following parameters: risk score, clinical stage, TNM stage, age, and sex. A calibration curve of the nomogram model was established to assess the consistency between the predicted and observed results.

Gene Set Enrichment Analysis (GSEA) used predefined gene sets (gmt files C2 and C5) to rank genes according to their differential expression levels in the two risk groups using the clusterProfiler R package. Only items with a *P*-value < 0.05 were considered. GSEA was conducted to normalize the gene expression profile and to excavate GO and KEGG pathways.

### Imaging protocol

In the radiogenomics section, the study initially included 245 patients, all of whom underwent preoperative abdominal CT or MRI, with ccRCC from TCGA-KIRC database. The patients underwent standard three-phase scans, including the cortical phase (25–30 s after contrast injection), parenchymal phase (60–70 s after contrast injection), and secretory phase (2–3 min after contrast injection). Iodine contrast injection standards were obtained from TCIA database for each hospital reference standard. The inclusion criteria were as follows: (1) confirmation of ccRCC with TNM staging obtained based on postoperative pathology; (2) preoperative contrast-enhanced CT (CECT) scan imaging data were complete, and a standard kidney CT triple enhancement scan protocol was used; and (3) recognizable mass lesions that could be detected in the kidney by parenchymal images with CE-CT scans. Exclusion criteria included the following: (1) a dissatisfactory quality of the CE-CT scans or the presence of large artifacts influencing the judgment of the lesion area; (2) radiomic features that cannot be successfully extracted through CECT scans. The detailed procedures of our study are shown in **Figure S1**.

### Image preprocessing and region-of-interest acquisition

Based on previous studies on kidney-associated radiomics, we selected the parenchymal phase in CECT scans to extract the radiomics features most associated with immune heterogeneity (23, 24). In the process of image sketching, an imaging physician (with 15 years of work experience in diagnostic urologic CT imaging) identified and fragmented the lesion contours on each slice within the sequence using the 3D Slicer software (version 4.11.2; Boston, MA, USA). Features were then established using the radiomics extraction software Pyradiomics (3.0.0; https://github.com/Radiomics/pyradiomics). Following this, we processed the obtained feature data by utilizing the min–max approach.

Intra-class and inter-class correlation coefficients (ICC) were used to assess the stability of the acquired features. Fifty patients were randomly selected for repeat region-of-interest (ROI) fragmentation by both the previous radiologist and a new radiologist (with 8 years of experience in urologic CT imaging) 30 days after the initial segmentation. Both physicians were unaware of the history of kidney disease and pathological diagnosis of the patients.

### Preliminary construction of the radiogenomics biomarker

In TCGA-KIRC database, radiogenomics features extracted from CECT images were filtered, and radiogenomics models were constructed according to the following sequential steps. Firstly, features with both intra-class and inter-class correlation coefficients greater than 0.75 were allowed as components of the potential immune-related radiogenomics model. The minimum redundancy-maximum relevance (mRMR) method was used for further feature dimensionality reduction with sufficient stability. LASSO analysis was then used for the selection of optimal radiogenomics features. The selected optimal features were linearly combined with pass-through coefficients to construct radiogenomics labels associated with immune-related gene expression levels, also known as RADscores. Evaluation of the novel radiogenomics biomarker was performed using the Mann–Whitney U test, mainly to classify patients into high- or low-risk groups based on immune-related gene expression. ROC curves were calculated to assess the predictive efficacy of the preliminary radiogenomics model.

### Nomogram construction based on the radiogenomics model

To further increase the credibility of the model, clinical and pathological information from the TCIA-KIRC database was combined in the radiogenomics model. Univariate analysis was performed to screen for elements associated with altered genetic subsets in the tumor microenvironment. In the training cohort, variables with P < 0.1 in the univariate regression were subsequently assigned to the multivariate logistic regression. A clinical model was constructed to include factors with P < 0.1 in the previous step of the multivariate analysis; backward stepwise selection was performed using the likelihood ratio test. We also compared the predictive power of the preliminary radiogenomics marker with clinical models for tumor immune-related gene risk model stratification. Finally, a combined multivariate logistic model was constructed using the RADscores and the selected clinicopathological factors. Variance inflation factor (VIF) analysis was performed on the combined model to further reduce the probability of overfitting. A nomogram was developed to visualize the final radiogenomics model, specifically to score each patient and quantify the levels of immune-related gene expression.

### Radiogenomics biomarker validation

The combined model was evaluated in the patient cohort, including the training and testing groups. ROC curves and AUC values were adopted to assess the predictive recognition capability of the immune microenvironment in the combined model. Calibration curves and Hosmer–Lemeshow tests were used to estimate the agreement between the predicted outcomes and the expected probabilities in the radiogenomics model.

We also used decision curve analysis (DCA) to analyze the clinical potential of the radiogenomics biomarker and calculated the net benefit of the model for different threshold probabilities.

### Statistical analysis

All statistical analyses were performed using the R software (version 3.6.3). The “survivalROC” package was employed to calculate the area under curve (AUC) of the ROC curve to assess the clinical utility of the prognostic model for clinical outcome. Kaplan–Meier (KM) analysis was conducted to assess survival differences among subtypes, with overall survival (OS) as the primary outcome. Disease-specific survival (DSS) and progression-free survival (PFS) were calculated as secondary outcomes. Continuous data were evaluated using the Wilcoxon rank-sum test. In addition, Fisher’s exact test was used to calculate differences in categorical data. Statistical significance was set at *P* value < 0.1.

## Results

### Identification of molecular subtypes

**Figure 1:**
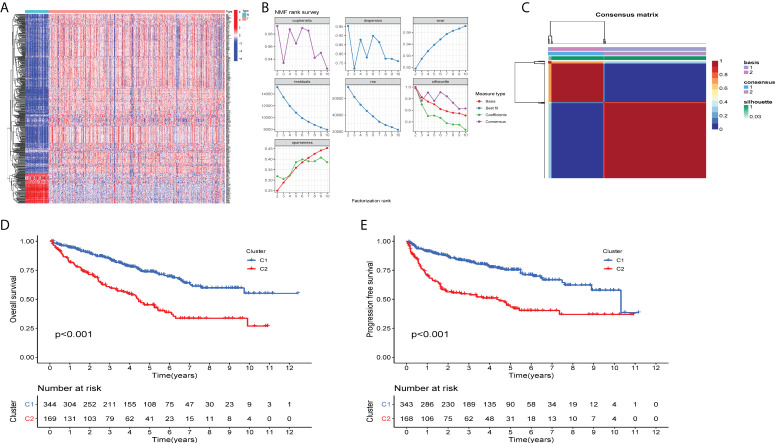
Two distinct TME-related molecular subtypes were identified by NMF analysis for ccRCC **(A)** The heatmap displays the expression patterns of TME-related genes in the tumors and normal tissues. N is equal to normal tissues, T is equal to tumor tissues. **(B)** Factorization rank for k = 2–10. **(C)** The heatmap of the consensus matrix when the consensus clustering k = 2. The value range is 0–1. The columns and rows are sorted through hierarchical clustering according to the Euclidean distance of the average link. **(D)** Kaplan–Meier OS curves and **(E)** PFS curve for the two clusters in TCGA-ccRCC dataset. The assessment of difference was achieved by log-rank test.

To investigate the molecular subtypes of ccRCC, transcriptomic data of ccRCC patients from TCGA database were retrieved. The expression information of tumor microenvironment-related genes was extracted. The heatmap displayed a different distribution of TME-related genes between tumor tissues and normal tissues in ccRCC (**Figure 1A**). The NMF algorithm was used to cluster patients into diverse subtypes according to TME gene expression levels. To ensure robust clustering results, the cophenetic correlation coefficient was used to determine the optimal number of clusters, and K = 2 was selected as the optimal cluster number after comprehensive consideration (**Figure 1B**). When k = 2, we observed that the two subtypes (C1 and C2) had clear boundaries, indicating that the ccRCC samples had stable and reliable clustering (**Figure 1C**). The survival curve (**Figures 1D, E**) showed that the overall survival of cluster 1 was significantly better than that of cluster 2 (*P* < 0.001). In addition, C1 had a significant advantage in progression-free survival compared to C2 (*P* < 0.001).

### Relationship between molecular subtypes and the tumor microenvironment

**Figure 2:**
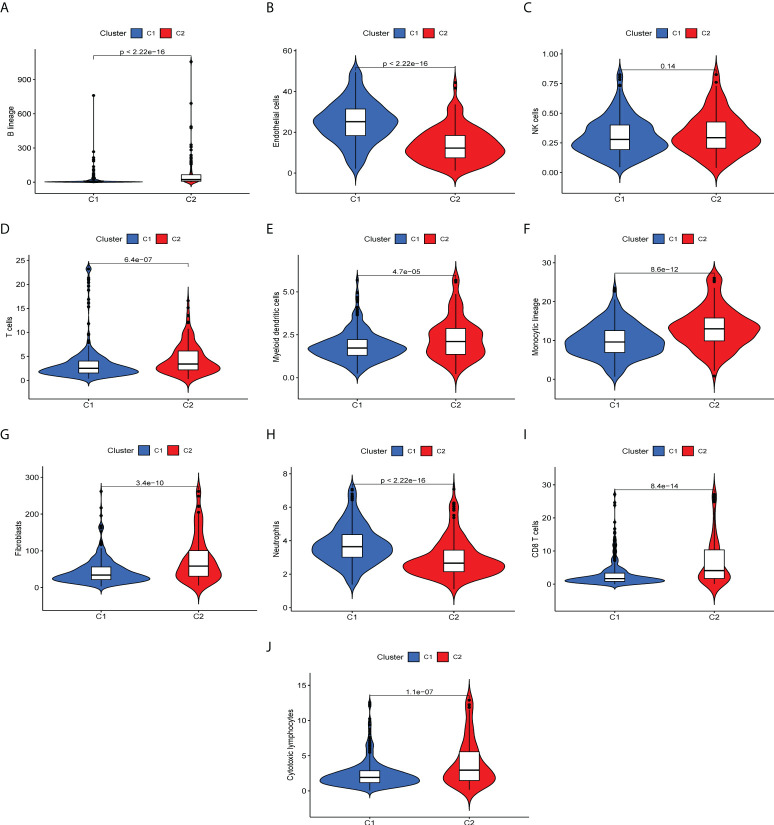
Differences of immune infiltrating cells in the immune microenvironment of two ccRCC molecular subtypes. **(A-I)** Levels of immune cell infiltration in the two subtypes. **(A)** B lineage, **(B)** endothelial cells, **(C)** NK cells, **(D)** T cells, **(E)** myeloid cells, **(F)** monocytic lineage, **(G)** fibroblasts, **(H)** neutrophils, **(I)** CD 8+T cells, **(J)** cytotoxic lymphocytes.

To determine immune-related gene expression in the tumor microenvironment, we explored immune cell infiltration in the two ccRCC subtypes using the MCPcount algorithm. The degree of invasion of the 10 immune cell populations in each ccRCC patient was evaluated, as shown in **Figure 2**. Ten immune cell groups were variable between the two subtypes. The levels of T cells, myeloid dendritic cells, monocyte lineage cells, fibroblasts, and cytotoxic lymphocytes were significantly increased in cluster 2. In contrast, the numbers of endothelial cells and neutrophils were decreased in cluster 1. For each type of immune cell expression data, the FDR values (q-values) for statistical differences between the two groups are available in the supplementary material.

### Identification of prognostic features in renal clear cell carcinoma

To further investigate the quantification of TME indicators for individual ccRCC patients, we performed in-group validation using TCGA datasets. The patients were divided into training and validation groups on a 7:3 scale. Univariate Cox regression analysis was performed to select genes with prognostic significance; 245 significant prognostic genes were retained. LASSO and Cox regression analyses were then performed to reduce redundancy. Fourteen genes were identified. Finally, in the prognostic model constructed by the multivariate regression method, a total of eight risk genes were selected, including PIMREG, CXCL5, UCN, KRBA1 PABPC1L RNASE2 IL4I1, and ABCB4. We then constructed a risk score formula based on the expression of specific genes and the coefficients calculated by multivariate Cox regression as follows: risk score = 0.461*PIMREG+0.11*CXCL5+0.526*UCN-0.465*KRBA1+0.200*PABPC1L+0.254*RNASE2+0.261*IL4I1-0.595*ABCB4.

**Figure 3:**
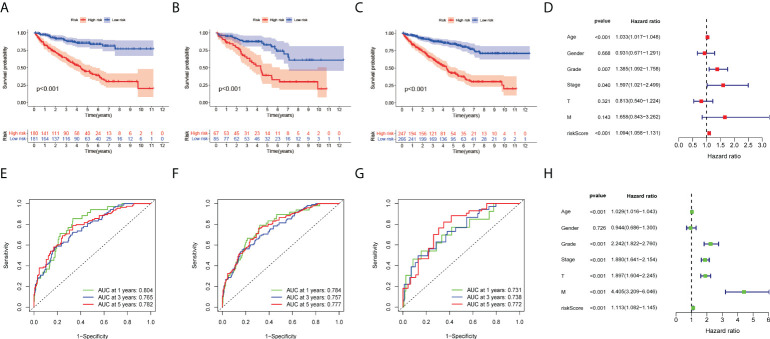
Development of eight risk genes for ccRCC. **(A-C)** Kaplan–Meier OS curve for high- and low-risk groups. **(A)** Training group, **(B)** validation group, and **(C)** TCGA dataset. **(D)** Forest plots displayed the univariate Cox regression analysis results of the risk score and clinical factors with OS. **(E-G)** Receiver operating characteristic curves (ROC curves) for the 1-, 3- and 5-year OS periods. **(E)** Training group, **(F)** validation group, and **(G)** TCGA dataset. **(H)** Forest plots display the multivariate Cox regression analyses results of the risk score and clinical factors with OS.

We further investigated the clinical outcomes of high-risk and low-risk patients using a risk prognostic model. Kaplan–Meier curves exhibited lower overall survival (OS) in the high-risk group in the training, validation, and TCGA datasets (*P* < 0.001, **Figures 3A–C**). We further investigated the clinical outcomes of patients with high or low expression of these eight risk genes alone as genomic markers through Kaplan–Meier analysis of OS, and the specific results are presented in the supplementary material. It should be noted that, although individual genes also have good survival prediction, the immune-related genetic risk model we constructed was able to significantly improve the predictive efficacy of survival stratification. We also adopted other survival times (including DSS and PFS) as the survival evaluation method, demonstrating the predictive value of our immune-related genetic risk model for the clinical prognosis of ccRCC patients.

### Validation of prognostic features in ccRCC

**Figure 4:**
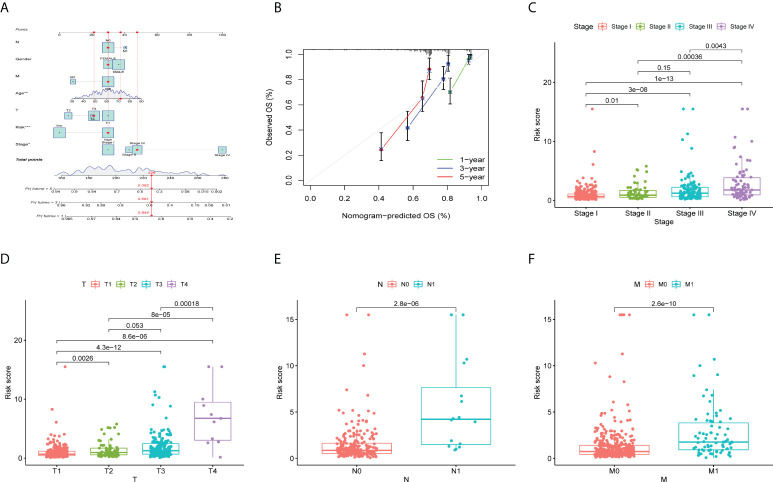
Validation of prognostic features in ccRCC. **(A)** Construction of a nomogram combining the risk prognostic signature and clinical features for prediction of OS. **(B)** Calibration plots display the actual and nomogram-predicted probability of the 1-, 3- and 5-year OS periods. **(C-F)** The box plot depicts the relationship between risk score and clinicopathology. **(C)** N stage, **(D)** M stage, **(E)** clinical stage, and **(F)** T stage. *P < 0.05; **P < 0.01; ***P < 0.001.

**Figure 5:**
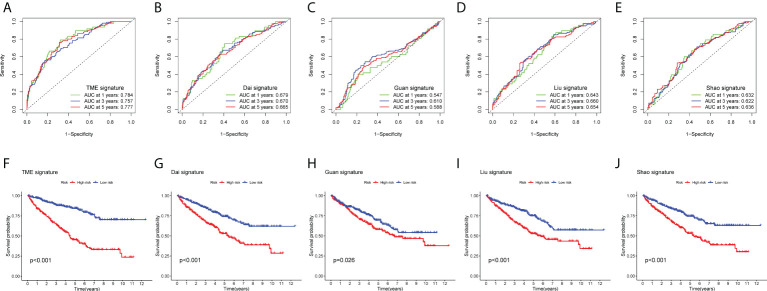
Comparison between prognostic models. **(A–E)** Receiver operating characteristic curves (ROC curves) for the 1-, 3-, and 5-year OS periods. **(A)** TME-related risk prognostic model, **(B)** Dai signature, **(C)** Guan signature, **(D)** Liu signature, and **(E)** Shao signature. **(F–J)** Kaplan–Meier OS curve for high- and low-risk groups. **(F)** TME-related risk prognostic model, **(G)** Dai signature, **(H)** Guan signature, **(I)** Liu signature, and **(J)** Shao signature.

To evaluate the clinical applicability of the risk prognostic model as a tool to predict the survival probability of patients with renal clear cell carcinoma, receiver operating characteristic curve (ROC) analysis was performed. The area under the curve (AUC) values of the 1-year OS in the training, validation, and TCGA datasets were 0.804, 0.731, and 0.784, respectively. The AUC values of 3-year OS were 0.765, 0.738, and 0.757, respectively. The AUC values of 5-year OS were 0.782, 0.772, and 0.777, respectively (**Figures 3E–G**). Then, univariate Cox regression and multivariate Cox regression analyses demonstrated that the risk prognostic model was an independent predictive biomarker (**Figures 3D, H**). In addition, to improve the clinical applicability of the risk prognostic model, we constructed a nomogram, including TNM stage, sex, age, and clinical stage (**Figure 4A**). The calibration diagram shows a fair agreement between the prediction results and the actual observation results of the nomogram model (**Figure 4B**). In addition, we compared the advantages and disadvantages of the TME prognostic risk model and other reported prognostic risk models, including models constructed in previous studies. The AUC values of 5-year OS were all higher than those of other known risk models (25–28) (**Figures 5A–E**). The K-M curve indicated that our immune-related prognostic risk model had the highest differentiation for patients’ clinical outcomes (**Figures 5F–J**).

### Comparison of clinicopathological features in high- and low-risk groups

We investigated the clinicopathological features of patients in the high-risk and low-risk groups, including T stage, N stage, lymphatic metastasis, and distant metastasis. The higher the clinical stage and T stage (**Figures 4C, D**), the more extensive the tumor lesion, and the higher the risk score (*P* < 0.05). In addition, there was a positive correlation between clinical stage and risk score (*P* < 0.05). Patients with lymphatic or distant metastasis had a higher risk factor (*P* < 0.1) (**Figures 4E, F**).

### Preliminary radiogenomics biomarker construction and evaluation

**Figure 6:**
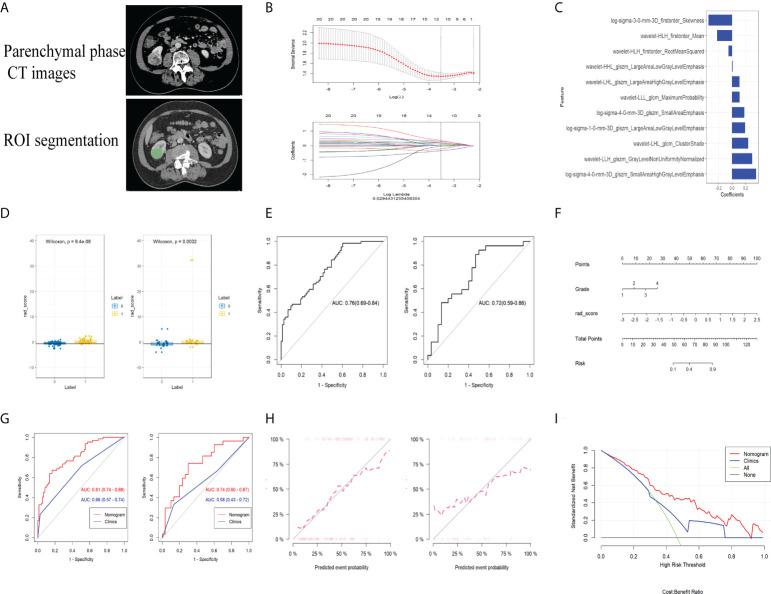
**(A)** CT image phase selection and ROI segmentation. **(B)** LASSO regression applied in the radiomics feature screening process. **(C)** The radiomics features finally screened for building the radiogenomics model. **(D)** M-W test showing that the radiomics model can effectively distinguish between the established gene subsets in both the training and validation groups. **(E)** ROC curves of the predictive power of the pure radiomics model for gene subsets in both the training and validation groups. **(F)** Nomogram built by combining radiomics as well as clinical and pathological information. **(G)** Predictive performance of the nomogram in both training and validation groups. **(H)** Calibration curves showing excellent fitting ability of the established radiogenomics model. **(I)** DCA curves of the established radiogenomics nomogram compared with the clinical model.

The CT images of 193 patients corresponding to the above transcriptomic data were included based on defined criteria for image collection (poor image quality or failure to identify the area of lesion by the imaging physicians) in TCIA-KIRC database (**Figure 6A**). From the enhanced CT scans of all patients, 1,218 features were extracted separately. After ICC evaluation, a minimum criterion of 0.75 for intra- or inter-ICC values was applied, leaving 821 features for initial feature screening. The most influential 30 features were then retained using the mRMR algorithm. Thirteen radiomics features were selected using LASSO regression (**Figure 6B**) to construct a preliminary radiogenomics model; the specific features are shown in **Figure 6C**. The formula for RADscores is shown in Supplementary Material II.

From the Mann–Whitney U test (**Figure 6D**), there was a significant difference in expression between the subsets of tumor microenvironment gene composition in the training cohort (*P* < 0.01), which was confirmed in the independent validation cohort (*P* < 0.05). The AUC values of the preliminary radiogenomics model were 0.76 in the training cohort and 0.72 in the validation cohort (**Figure 6E**).

### Nomogram construction based on the radiogenomics model

In the univariate analysis of the clinical model building, only grade was significantly associated with tumor microenvironment gene subset grouping (*P* < 0.1). It retained statistical significance in the multivariate logistic regression analysis (*P* < 0.1) and therefore constituted the clinical model. The combined model was constructed by combining RADScore and Grade. Finally, we visualized this ultimate radiogenomics marker in the form of a nomogram, as shown in **Figure 6F**.

### Final radiogenomics model validation and clinical use evaluation

The radiogenomics biomarker based on the subset of tumor immune-related gene expression showed good predictive performance (AUC of 0.81 and 0.74 in the training and validation cohorts, respectively) in reflecting tumor immune-related gene expression alterations (**Figure 6G**). The Delong test showed a statistically significant difference in AUC values between the combined nomogram and the clinical model (*P* < 0.01). The calibration curves (**Figure 6H**) showed good agreement between the predicted and the observed probabilities of the combined nomograms.

Decision curve analysis showed that the radiogenomics nomogram provided a net benefit compared to the “treat all” or “no treatment” strategy with a threshold probability of more than 10% for the clinical model (**Figure 6I**). This indicates that the radiogenomics nomogram has an excellent clinical utility.

## Discussion

In this study, a novel radiogenomics biomarker was constructed to predict the prognosis of patients with ccRCC. Such a biomarker was built on its close relationship with immune-related gene expression detected by transcriptomic analysis in patients with ccRCC. Survival statistics demonstrated that it could effectively stratify the prognosis of ccRCC patients. We aim to improve the process of precise tumor diagnosis and treatment through radiogenomics and to promote the deep investigation and mining of genetic information by imaging methods.

As solid tumors exhibit completely different drug therapeutic efficacies and disease progression in different patients, the tumor microenvironment is increasingly becoming a scientific hotspot for research on therapeutic resistance of tumors as well as target selection. In clear cell renal cell carcinoma, large-scale genomic studies have identified somatic mutations that affect tumor progression and the response to immune checkpoint blockade therapy (29–31). Unfortunately, despite the large number of genetic markers currently constructed for the tumor microenvironment of kidney cancer and the good results they have achieved in prognosis prediction, their reliability and practical clinical application remain unconvincing; there are still major obstacles facing the use of multiple genetic markers in practical clinical application. Therefore, it is extremely important to identify the infiltration of TME and immune-related gene expression in ccRCC patients using non-invasive diagnostic methods.

Radiomics is different from the conventional perspective of image information interpretation, which uses high-level algorithms and advanced image processors (32–34). The fundamental problems facing radiomics toward clinical applications, such as the poor interpretability of extracted features and the inability of more advanced deep learning methods to explain them in a completely “black box,” have not received much attention from researchers. In our study, we found that a radiogenomics marker based on genetic information from the TME can reflect profound alterations in immune-related gene expression. Their expression levels are closely correlated with a variety of immune cells, such as T cells, myeloid dendritic cells, and fibroblasts. In addition, there were significant correlations between the alterations and pathways such as tumor fibrosis and microvascular infiltration. This suggests that the novel radiogenomics marker we constructed could adequately reflect the various profound modifications of the TME. Few previous studies have reported the application of radiogenomics approaches for the non-invasive monitoring of TME. This gives our study a unique advantage, although this research approach needs to be supported by further experimental data. However, it also provides a fresh and dynamic methodological guide for the optimal combination of imaging and genomic data. However, it must also be acknowledged that a direct association between the immune microenvironment of ccRCC and the clinical prognosis of patients has not been confirmed by large-scale clinical data. Therefore, our study was based on transcriptomic data, and it aimed to assess immune-related gene expression in tumors in a non-invasive manner. This way, prognostic differences in tumors can be explained from an immunological perspective. Notably, some ccRCC patients often suffer from a coexisting disease with abnormal autoimmune system function, which may have a confounding effect on the ultimate predictive efficacy of immune-based prediction models. We therefore recommend that immune-related clinical treatment decisions and predictive models be considered with caution in such patients. In our work, we have incorporated some valid clinical and pathological factors in addition to immune-related genes in the hope of diluting the adverse effects on model prediction in this subset of patients. However, specific predictive efficacy requires future clinical validation of transcriptomic data and imaging for this subset of immune abnormal ccRCC patients.

Some limitations exist in this study: (1) the images in the study were obtained from TCIA database, utilizing different imaging machines and image acquisition protocols. Although strict inclusion and exclusion criteria were used, the results need to be validated in future clinical trials in more centers. (2) Robust data on radiogenomics and specific phenotypes of the tumor microenvironment are still lacking, and we hope to deepen our understanding of this in the future by combining conventional methods with radiogenomics-based methods. (3) Concerning the comprehensiveness of clinical information in TCGA database, only select clinical and pathological factors were included in the radiogenomics model, and more factors, such as conventional markers, may be needed in the future to improve the predictive power and reliability of the model. (4) Radiomics analysis was based on the ROI of the entire tumor, while the biopsy was tumor-specific. Owing to the spatial resolution of the CT image and the unavailability of tumor sampling location information, it has not yet been possible to achieve a more accurate prediction between the two correspondences. We hope to make further breakthroughs on this problem in future work.

In conclusion, this study constructed a novel non-invasive radiogenomics marker for the prognostic stratification of ccRCC. Based on the contrast-enhanced CT scans and radiogenomics features in ccRCC patients, this biomarker achieved convergent prediction of immune-related gene risk model stratification and pathway alterations. Such a novel imaging-based approach, used to reveal tumor microenvironment alterations, may have great clinical value for future immunotherapy efficacy and individualized tumor treatment.

## Data availability statement

The datasets presented in this study can be found in online repositories. The names of the repository/repositories and accession number(s) can be found in the article/**Supplementary Material**.

## Ethics statement

This study was reviewed and approved by The institute review board of Huashan hospital, Fudan University. The patients/participants provided their written informed consent to participate in this study. Written informed consent was obtained from the individual(s) for the publication of any potentially identifiable images or data included in this article.

## Author contributions

JG, FY, and FH contributed equally to this work. Research establishment, JG and FY. ROI outlining and image filtering, FH. Data analysis, JG. Supervision, JZ and HJ. All authors contributed to the article and approved the submitted version.

## Funding

This work was funded by the National Key Research and Development Project in China (No. 2017YFC0113405), National Natural Science Foundation of China (82071877), and Natural Science Basic Research Plan of Shaanxi (No. 2022JQ-811).

## Conflict of interest

The authors declare that the research was conducted in the absence of any commercial or financial relationships that could be construed as a potential conflict of interest.

## Publisher’s note

All claims expressed in this article are solely those of the authors and do not necessarily represent those of their affiliated organizations, or those of the publisher, the editors and the reviewers. Any product that may be evaluated in this article, or claim that may be made by its manufacturer, is not guaranteed or endorsed by the publisher.

## References

[1] Siegel RL Miller KD Fuchs HE Jemal A. Cancer statistics, 2021. CA: Cancer J Clin (2021) 71(1):7–33. doi: 10.3322/caac.21654 33433946

[2] Sung H Ferlay J Siegel RL Laversanne M Soerjomataram I Jemal A. Global cancer statistics 2020: GLOBOCAN estimates of incidence and mortality worldwide for 36 cancers in 185 countries. CA: Cancer J Clin (2021) 71(3):209–49. doi: 10.3322/caac.21660 33538338

[3] Wettersten HI Aboud OA Lara PNJ Weiss RH. Metabolic reprogramming in clear cell renal cell carcinoma. Nat Rev Nephrol (2017) 13(7):410–9. doi: 10.1038/nrneph.2017.59 28480903

[4] Schödel J Grampp S Maher ER Moch H Ratcliffe PJ Russo P. Hypoxia, hypoxia-inducible transcription factors, and renal cancer. Eur Urol (2016) 69(4):646–57. doi: 10.1016/j.eururo.2015.08.007 PMC5012644 26298207

[5] Krishna C DiNatale RG Kuo F Srivastava RM Vuong L. Single-cell sequencing links multiregional immune landscapes and tissue-resident T cells in ccRCC to tumor topology and therapy efficacy. Cancer Cell (2021) 39(5):662–77. doi: 10.1016/j.ccell.2021.03.007 PMC8268947 33861994

[6] Turajlic S Xu H Litchfield K Rowan A Chambers T Lopez JI. Tracking cancer evolution reveals constrained routes to metastases: TRACERx renal. CELL (2018) 173(3):581–94. doi: 10.1016/j.cell.2018.03.057 PMC5938365 29656895

[7] Yong C Stewart GD Frezza C. Oncometabolites in renal cancer. Nat Rev Nephrol (2020) 16(3):156–72. doi: 10.1038/s41581-019-0210-z PMC7030949 31636445

[8] Huang WC Donin NM Levey AS Campbell SC. Chronic kidney disease and kidney cancer surgery: New perspectives. J Urol (2020) 203(3):475–85. doi: 10.1097/JU.0000000000000326 31063051

[9] Kats-Ugurlu G Oosterwijk E Muselaers S. Neoadjuvant sorafenib treatment of clear cell renal cell carcinoma and release of circulating tumor fragments. Neoplasia (New York, N.Y.) (2014) 16(3):221–8. doi: 10.1016/j.neo.2014.03.007 PMC4094826 24726142

[10] Rini BI Plimack ER Stus V Gafanov R. Pembrolizumab plus axitinib versus sunitinib for advanced renal-cell carcinoma. N Eng J Med (2019) 380(12):1116–27. doi: 10.1056/NEJMoa1816714 30779529

[11] Motzer RJ Penkov K Haanen J Rini B Albiges L. Avelumab plus axitinib versus sunitinib for advanced renal-cell carcinoma. N Eng J Med (2019) 380(12): 1103–15. doi: 10.1056/NEJMoa1816047 PMC6716603 30779531

[12] Motzer RJ Robbins PB Powles T Albiges L Haanen JB Larkin J. Avelumab plus axitinib versus sunitinib in advanced renal cell carcinoma: biomarker analysis of the phase 3 JAVELIN renal 101 trial. Nature Med (2020) 26(11):1733–41. doi: 10.1038/s41591-020-1044-8 PMC8493486 32895571

[13] Dagogo-Jack I Shaw AT. Tumour heterogeneity and resistance to cancer therapies. Nat Rev Clin Oncol (2018) 15(2):81–94. doi: 10.1038/nrclinonc.2017.166 29115304

[14] Pe'er D Ogawa S Elhanani O Keren L Oliver TG Wedge D. Tumor heterogeneity. Cancer Cell (2021) 39(8):1015–7. doi: 10.1016/j.ccell.2021.07.009 34375606

[15] Chen X Song E. Turning foes to friends: targeting cancer-associated fibroblasts. Nat Rev Drug Discov (2019) 18(2):99–115. doi: 10.1038/s41573-018-0004-1 30470818

[16] Chen Y McAndrews KM Kalluri R. Clinical and therapeutic relevance of cancer-associated fibroblasts. Nat Rev Clin Oncol (2021) 18(12):792–804. doi: 10.1038/s41571-021-00546-5 34489603 PMC8791784

[17] Gossage L Eisen T Maher ER. VHL, the story of a tumour suppressor gene. Nat Rev Cancer (2015) 15(1):55–64. doi: 10.1038/nrc3844 25533676

[18] Choueiri TK Kaelin WGJ. Targeting the HIF2-VEGF axis in renal cell carcinoma. Nat Med (2020) 26(10):1519–30. doi: 10.1038/s41591-020-1093-z 33020645

[19] Moussa AM Ziv E. Radiogenomics in interventional oncology. Curr Oncol Rep (2021) 23(1):9. doi: 10.1007/s11912-020-00994-9 33387095

[20] Badic B Tixier F Cheze Le Rest C Hatt M Visvikis D. Radiogenomics in colorectal cancer. Cancers (2021) 13(5):973–81. doi: 10.3390/cancers13050973 PMC7956421 33652647

[21] Meng Y Cai K Zhao J Huang K Ma X Song J. Transcriptional profiling reveals kidney neutrophil heterogeneity in both healthy people and ccRCC patients. J Immunol Res (2021) 2021:5598627. doi: 10.1155/2021/5598627 33791390 PMC7984911

[22] Ursprung S Beer L Bruining A Woitek R Stewart GD Gallagher FA. Radiomics of computed tomography and magnetic resonance imaging in renal cell carcinoma-a systematic review and meta-analysis. Eur Radiol (2020) 30(6):3558–66. doi: 10.1007/s00330-020-06666-3 PMC7248043 32060715

[23] Rallis KS Kleeman SO Grant M Ordidge KL Sahdev A Powles T. Radiomics for renal cell carcinoma: Predicting outcomes from immunotherapy and targeted therapies-a narrative review. Eur Urol Focus (2021) 7(4):717–21. doi: 10.1016/j.euf.2021.04.024 33994170

[24] Suarez-Ibarrola R Basulto-Martinez M Heinze A Gratzke C Miernik A. Radiomics applications in renal tumor assessment: A comprehensive review of the literature. Cancers (2020) 12(6):1387–96. doi: 10.3390/cancers12061387 PMC7352711 32481542

[25] Dai S Zeng H Liu Z Jin K. Intratumoral CXCL13(+)CD8(+)T cell infiltration determines poor clinical outcomes and immunoevasive contexture in patients with clear cell renal cell carcinoma. J Immunother Cancer (2021) 9(2):1823–34. doi: 10.1136/jitc-2020-001823 PMC7887366 33589528

[26] Peng R Wang Y Mao L Fang F Guan H. Identification of core genes involved in the metastasis of clear cell renal cell carcinoma. Cancer Manag Res (2020) 12:13437–49. doi: 10.2147/CMAR.S276818 PMC7779301 33408516

[27] Chang K Yuan C Liu X. Ferroptosis-related gene signature accurately predicts survival outcomes in patients with clear-cell renal cell carcinoma. Front Oncol (2021) 11:649347. doi: 10.3389/fonc.2021.649347 33996565 PMC8120155

[28] Xu C Chen L Li D Chen F Sha M Shao Y. Acyl-CoA thioesterase 8 and 11 as novel biomarkers for clear cell renal cell carcinoma. Front Genet (2020) 11:594969. doi: 10.3389/fgene.2020.594969 33362855 PMC7758486

[29] Miao D Margolis CA Gao W. Genomic correlates of response to immune checkpoint therapies in clear cell renal cell carcinoma. Sci (New York N.Y.) (2018) 359(6377):801–6. doi: 10.1126/science.aan5951 PMC6035749 29301960

[30] Clark DJ Dhanasekaran SM Petralia FZ. Integrated proteogenomic characterization of clear cell renal cell carcinoma. Cell (2019) 179(4):964–83. doi: 10.1016/j.cell.2019.10.007 PMC7331093 31675502

[31] Au L Hatipoglu E Robert de Massy M Turajlic S. Determinants of anti-PD-1 response and resistance in clear cell renal cell carcinoma. Cancer Cell (2021) 39(11):1497–518. doi: 10.1016/j.ccell.2021.10.001 PMC8599450 34715028

[32] Mayerhoefer ME Materka A Langs G Häggström I Szczypiński P Gibbs P. Introduction to radiomics. J Nucl Med (2020) 61(4):488–95. doi: 10.2967/jnumed.118.222893 PMC9374044 32060219

[33] Fiz F Viganò L Gennaro N Costa G La Bella L Boichuk A. Radiomics of liver metastases: A systematic review. Cancers (2020) 12(10):2881–902. doi: 10.3390/cancers12102881 PMC7600822 33036490

[34] van Timmeren JE Cester D Tanadini-Lang S Alkadhi H Baessler B. Radiomics in medical imaging-"how-to" guide and critical reflection. Insights Imaging (2020) 11(1):91. doi: 10.1186/s13244-020-00887-2 32785796 PMC7423816

