# Supplementary material for: A radiogenomics biomarker based on immunological heterogeneity for non-invasive prognosis of renal clear cell carcinoma
Source: Front Immunol. 2022 Sep 13;13:956679. doi: 10.3389/fimmu.2022.956679 (PMC9513051; doi:10.3389/fimmu.2022.956679)
Supplement: Supplementary file 1 [file DataSheet_1.doc]

**Supplementary Material**

**I. Radiomics features extraction methodology**

**II.** **Calculation formula of the radiogenomics biomarker.**

**IIII****. R packages we used in this study**

**Figure. S1.** Recruitment pathway for patients in this study.

**Figure. S2.** The process of constructing immune-related genetic markers for tumor microenvironment based on the "NMF" method.

**Figure. S3.** Survival analysis of clinical outcomes in patients with high and low expression in the prognostic model characterized by 8 risk genes alone.

**Figure. S4.** Survival analysis of immune-related genetic risk models (DSS and PFS as survival evaluation criteria, respectively)

**Figure. S5.** Correlation of genomic markers with genes, immune cells and signaling pathways related to immune infiltration in tumor microenvironment based on "NMF" method.

**Table. S1.** The FDR values (q-values) for statistical differences between the two groups in each type of immune cell expression data.

245 patients with ccRCC were collected from TCIA-KIRC database who met the inclusion criteria

Finally,194 patients were enrolled in this study

20 patients with other malignancies in the kidney

26 patients excluded with blurring lesions due to artifacts or inflammation of kidney

5 patients whose imaging features cannot be extracted from the parenchymal phase of CE-CT scans

**Figure S1.** Recruitment pathway for patients in this study.

**
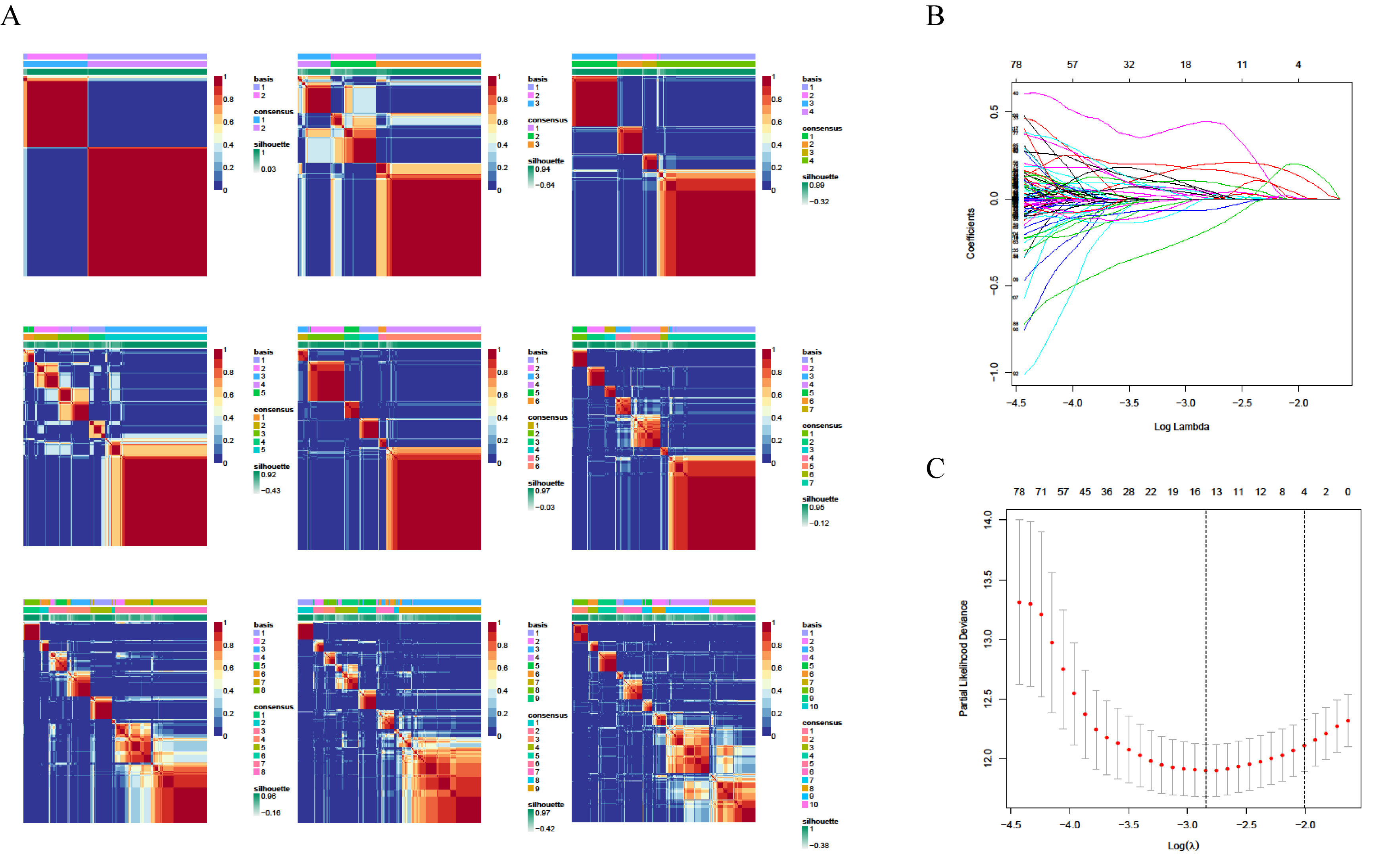
**

**Figure S2.** The process of constructing immune-related markers for tumor microenvironment based on the "NMF" method. (A) Screening of different gene subgroups by NMF method. (B) and (C) LASSO regression to construct genomic markers.


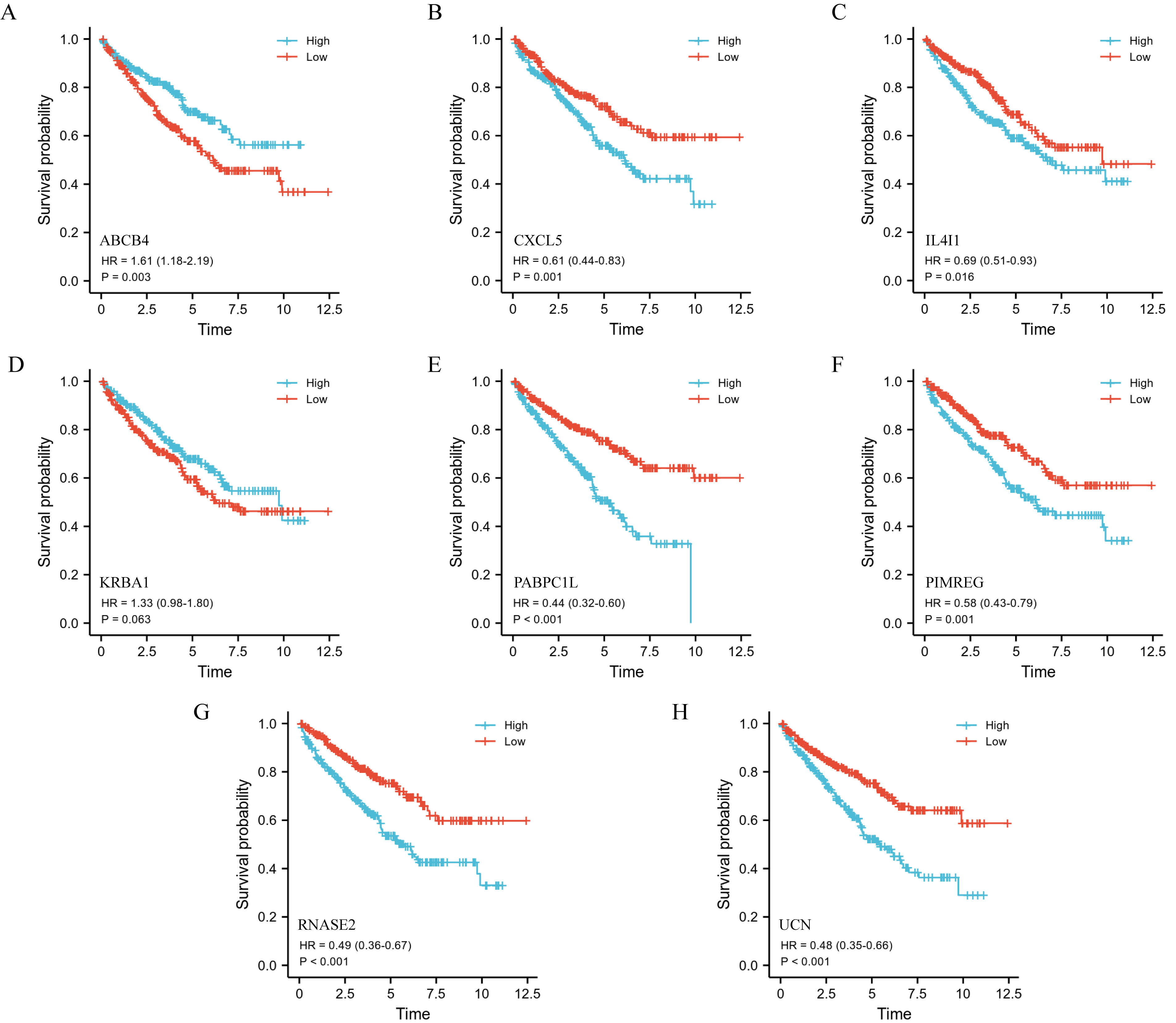


**Figure. S3.** Survival analysis of clinical outcomes in patients with high and low expression in the prognostic model characterized by 8 risk genes alone.


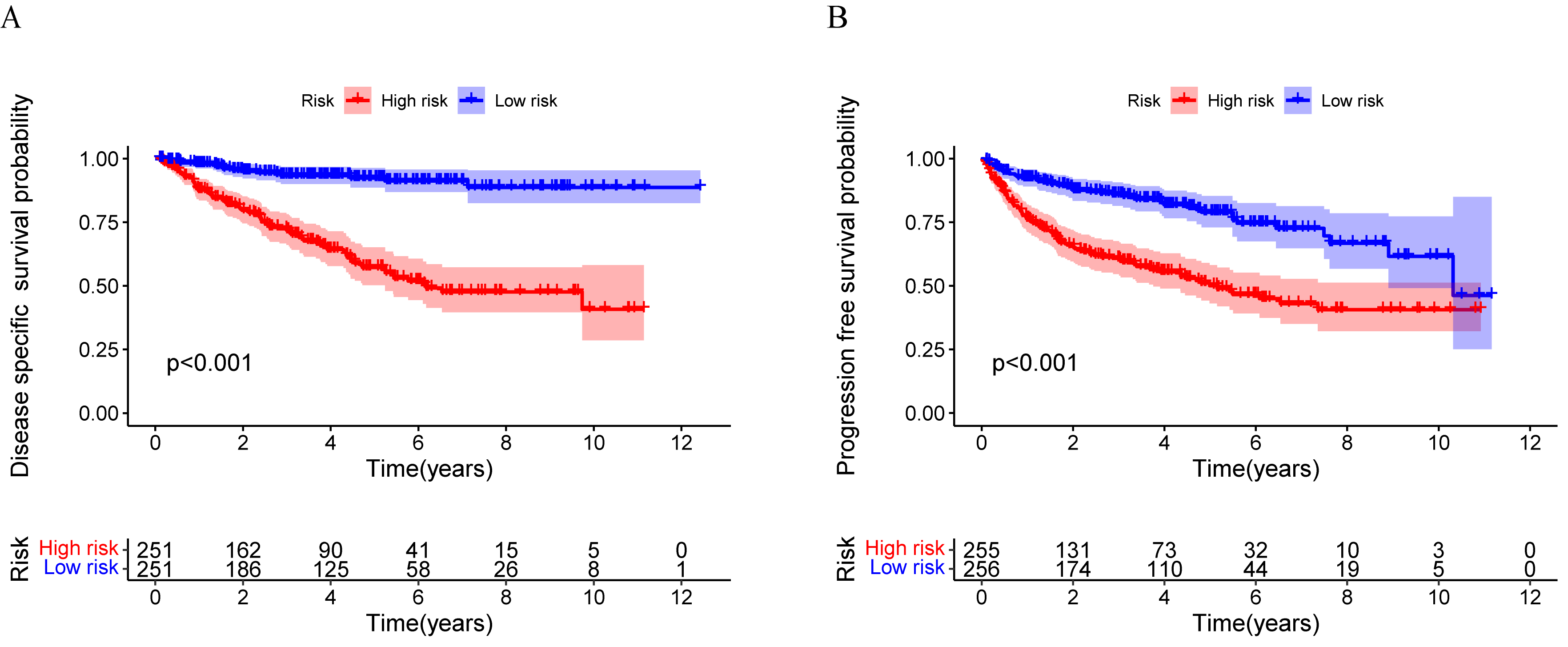


**Figure. S4.** Survival analysis of immune-related genetic risk model (DSS and PFS as survival evaluation criteria, respectively).


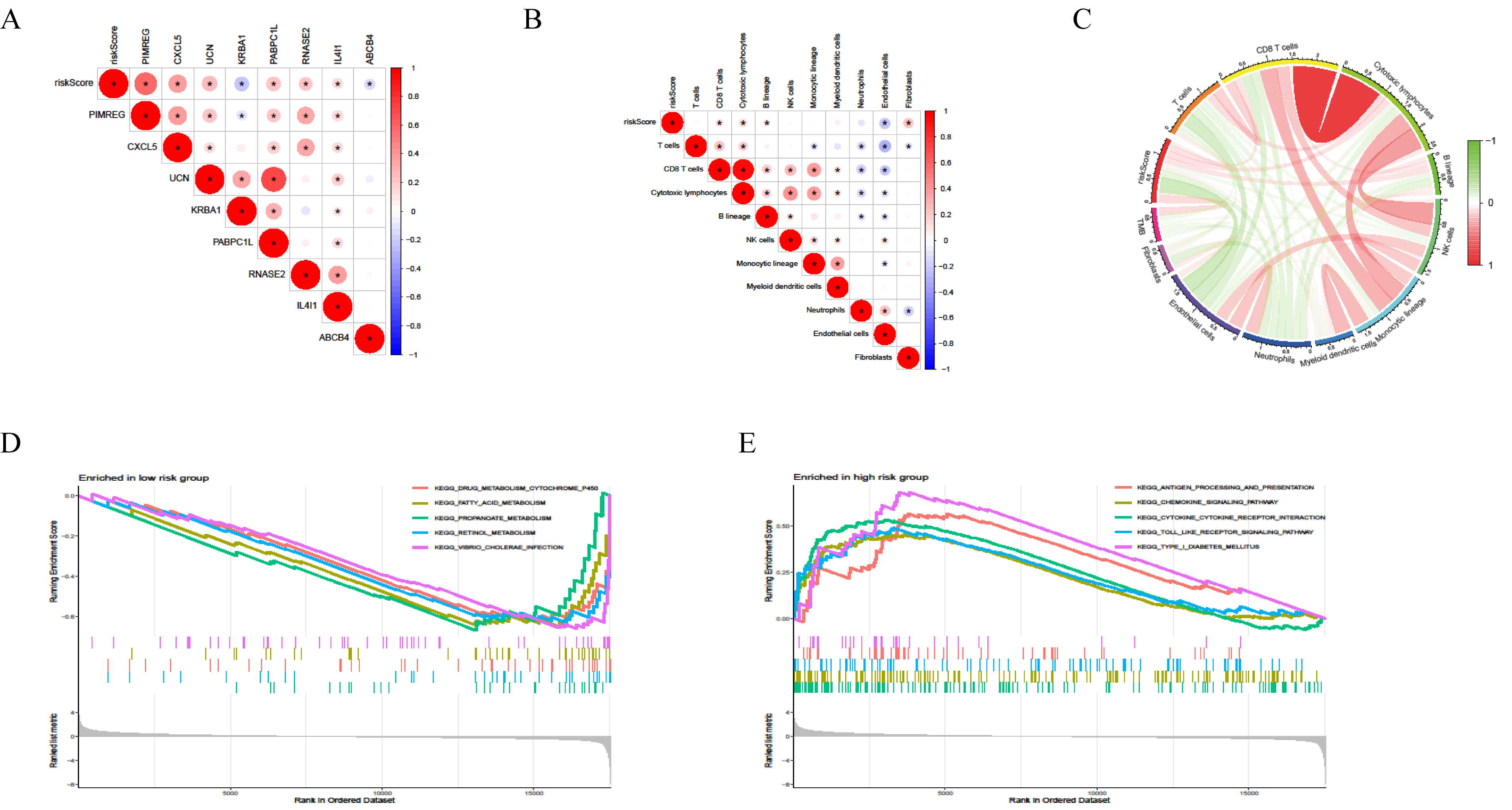


**Figure S5.** Correlation of genomic markers with genes, immune cells and signaling pathways related to immune infiltration in tumor micro-environment based on "NMF" method. (A) The relationship between Riskscore and tumor microe-nvironment immune-related genes, including PMREG, CXCL5, UCN, KRBA1, PABPC1L; (B) & (C) The relationship between Riskscore and tumor micro-environment immune-related cells, including T cells, CD8 T cells, NK cells, etc.; (D) & (E) The correlation between Riskscore and tumor micro-environment immune infiltration signaling pathways.

**Table. S1.** The FDR values (q-values) for statistical differences between the two groups in each type of immune cell expression data.

| **Cell Type** | **K** | **P-value** | **Q-value** |
| --- | --- | --- | --- |
| B lineage | 1 | 2.22×10^-16^ | 2.22×10^-15^ |
| Neutrophils | 2 | 2.22×10^-16^ | 1.11×10^-15^ |
| Endothelial cells | 3 | 2.22×10^-16^ | 7.40×10^-16^ |
| CD8 T cells | 4 | 8.40×10^-14^ | 2.10×10^-13^ |
| Monocytic lineage | 5 | 8.60×10^-12^ | 1.72×10^-11^ |
| NK cells | 6 | 3.40×10^-10^ | 5.67×10^-10^ |
| Fibroblasts | 7 | 3.40×10^-10^ | 4.86×10^-10^ |
| Cytotoxic lymphocytes | 8 | 1.10×10^-7^ | 1.38×10^-7^ |
| T cells | 9 | 6.40×10^-7^ | 7.11×10^-7^ |
| Myeloid dendritic cells | 10 | 4.70×10^-5^ | 4.70×10^-5^ |

**I. R****adiomic****s features extraction methodology**

Using the feature extraction tool, we extracted 1218 radiomic features in CT images of the renal parenchymal phase. These radiomics features can be classified into six categories: (1) Shape features (n=14); (2) First-order statistics (n=18); (3) Gray-level co-occurrence matrix (GLCM) features (n=22); (4) Gray-level run length matrix (GLRLM) features (n=16); (5) Gray-level size zone matrix (GLSZM) features (n=16); (6) Gray-level dependence matrix ( GLDM) (n=14). After 5 wavelet filter transforms and 8 Gaussian Laplace (LoG) transforms, all kinds of features except shape features are increased by 14 times (1+5+8). Therefore, the total number of features extracted in each stage is: 14*1 (shape features) + (18+22+16+16+14)*14=1218.

The source of each feature and the specific calculation method are labeled in the feature extraction software: https://pyradiomics.readthedocs.io/en/latest/features.html.

To further clarify the association between the extracted features and tumor immune heterogeneity, we performed further feature dimensionality reduction and screening by machine learning algorithms. First, the features were selected by the minimum redundancy maximum correlation (mRMR) algorithm to enter. After that, the 30 features with the highest correlation with the results and the lowest correlation with each other were selected using LASSO regression. Finally, radiogenomics features were constructed by LASSO logistic regression model. The radiogenomics biomarkers were composed of features with non-zero coefficients in the LASSO regression by a linear combination of their weighted coefficients.

**II. Calculation formula of the radiogenomics biomarker.**

***Radiogenomic-score***== -0.315*log-sigma-3-0-mm-3D_firstorder_Skewness+0.089*wavelet-HLL_glszm_LargeAreaLowGrayLevelEmphasis+0.212*wavelet-HHH_firstorder_Mean+0.185*log-sigma-4-0-mm-3D_glrlm_LongRunHighGrayLevelEmphasis+-0.097*wavelet-HLH_gldm_SmallDependenceHighGrayLevelEmphasis+0.13*wavelet-HHH_glszm_SmallAreaLowGrayLevelEmphasis+-0.178*wavelet-HHH_gldm_SmallDependenceLowGrayLevelEmphasis+0.18*wavelet-LLH_glszm_GrayLevelNonUniformityNormalized+0.12 *wavelet-HLL_firstorder_Mean+0.358*wavelet-LHL_glcm_ClusterShade+0.178*wavelet-LLL_gldm_LargeDependenceHighGrayLevelEmphasis + -0.165

Nomoscore = (Intercept)*-2.13417571297222+Grade*0.780319672462528+rad_score*1.59290911864063

**III.** **R packages we used in this study**

The "glmnet" package was used for LASSO logistic regression. The "rms" package was used for logistic regression analysis, calibration plots and VIF calculations. Calibration curves were built using bootstrap validation with 1,000 resamples. ROC curves and Delong tests were performed using the "pROC" package. hosmer-Lemeshow tests were performed with the "generalhoslem" package. The "dca.R" package was used for decision curve analysis.
